# Dimensionality Effects in FeGe_2_ Nanowires: Enhanced Anisotropic Magnetization and Anomalous Electrical Transport

**DOI:** 10.1038/s41598-017-05771-6

**Published:** 2017-08-02

**Authors:** Siwei Tang, Ivan Kravchenko, T. Z. Ward, Qiang Zou, Jieyu Yi, Cheng Ma, Miaofang Chi, Guixin Cao, An-Ping Li, David Mandrus, Zheng Gai

**Affiliations:** 1 State Key Laboratory of Powder Metallurgy, Central South University, Changsha, 410083 China; 2 Department of Materials Science and Engineering, University of Tennessee, Knoxville, TN 37996 USA; 3 Center for Nanophase Materials Sciences, Oak Ridge National Laboratory, Oak Ridge, TN 37831 USA; 4 Materials Science and Technology Division, Oak Ridge National Laboratory, Oak Ridge, TN 37831 USA

## Abstract

We report the synthesis of single-crystal iron germanium nanowires via chemical vapor deposition without the assistance of any catalysts. The assembly of single-crystal FeGe_2_ nanowires with tetragonal C16 crystal structure shows anisotropic magnetic behavior along the radial direction or the growth axial direction, with both antiferromagnetic and ferromagnetic orders. Single FeGe_2_ nanowire devices were fabricated using e-beam lithography. Electronic transport measurement in these devices show two resistivity anomalies near 250 K and 200 K which are likely signatures of the two spin density wave states in FeGe_2_.

## Introduction

The spin density wave (SDW) state is a low-energy ordered state that arises as a consequence of electron-electron interactions, for which the density of the conduction electron spins is spatially modulated. The SDW state has many similarities to other broken symmetry ground states of metals, such as superconductivity and charge density waves^1–4^. As a much-studied elemental antiferromagnet and widely adopted into modern techniques, Cr has a model quantum phase transition to an antiferromagnetic SDW^4–9^. Other than the interesting bulk behavior, electrical measurements on Cr thin films show surprising film-thickness-dependent behavior directly related to SDW formation and the presence of antiferromagnetic domains^7^. Led by application demands, dimensionality effects have been widely studied and have led to the discovery of new physical phenomena when the dimension of nanoparticles, nanowires, and thin films comes close to the magnetic characteristic length. In the past, these efforts were largely focused on tuning ferromagnetic materials^5, 9–12^. This is unsurprising since antiferromagnets show no net external magnetic moment making them difficult to manipulate and characterize. Recently, this has started to change as it was recognized that this insensitivity to magnetic fields give antiferromagnets an advantage in resisting the stray fields that limit high density data storage^13^, resistive switching^14, 15^, and spintronic^16, 17^ applications. The ability to identify, fabricate, and utilize low dimensional antiferromagnetic materials is at the forefront of these works.

Scientific interest in exploring iron digermanide has been sustained for many years due to its complex magnetism^18–22^. The intermetallic compound FeGe_2_ has a tetragonal C16-type cell (space group 14/mcm) with a = 5.908 Å and c = 4.955 Å. This material is somewhat unusual in that the distances between the nearest iron atoms in the basal plane (4.2 Å) and along the *c*-axis (2.48 Å, the same distance as in bulk iron) are very different. These structural properties are tightly bound to magnetic properties; the short interatomic Fe-Fe spacing along the *c*-axis can be expected to order ferromagnetically along the *c*-axis and antiferromagnetically in the basal plane where the interatomic Fe-Fe spacing is greater. The complexity of this structure-property relationship is seen in neutron diffraction results, where FeGe_2_ undergoes two phase transitions when cooling in a magnetic field^18, 23, 24^. At 289 K, the material transitions from a paramagnet to an incommensurate SDW state. Further cooling to 263 K produces a transition from the incommensurate SDW state to a spiral antiferromagnetic phase (commensurate SDW state). At the incommensurate to commensurate transition, the iron magnetic moments retain ferromagnetic order along the *c-*axis but the moments in the basal plane change from a helical structure to antiferromagnetic order. This transition is also accompanied by a slight increase in the electrical resistivity, as the formation of a new magnetic superzone leads to extra scattering of the charge carriers^18, 22^.

The significance of the structure-property relationship in FeGe_2_ described above thus makes it a promising subject for observing novel dimensionality effects. Although other magnetic iron germanide nanowires have been successfully prepared^25–28^, nanowires of tetragonal FeGe_2_, with its extremely large interatomic Fe anisotropy, have not been previously reported. In this work, we report the synthesis of single-crystal FeGe_2_ nanowires using a chemical vapor deposition method. Magnetic measurements of FeGe_2_ nanowire arrays show strongly anisotropic behavior. Temperature and field dependent magnetization and ac susceptibility measurements show the existence of both antiferromagnetism and ferromagnetism. Four-probe transport resistance measurements on individual wires show anomalies at ~250 K and ~200 K which may be associated with the magnetic transitions of the SDW in FeGe_2_.

## Results/Discussion

The chemical vapor deposition system has been chosen for the growth of the nanowires. FeCl_3_ was placed upstream as the precursor in the glass tube, and a germanium (001) wafer was used as the growth substrate. The heat was provided by the tube furnace while the pressure and flow rate were adjusted by the pump, flowmeter and a valve.

Optimizing process parameters, adding various catalysts, or modifying the reactant (precursor and substrate) were used to grow silicide nanowires in a controlled way^11, 29, 30^. There were limited reports in literature even for the synthesis of iron-germanium compounds bulk crystals^31^. In our experiment, we found three processes which played crucial roles in the successful growth of iron-germanium nanowires: (1) the decomposition rate of the precursor, (2) the material growth rate on the substrate around the nucleation points, (3) the reaction speed of the elements. Processes (1) and (2) were most easily controlled by the furnace temperature. Process (3) was mainly adjusted by the carrier pressure of the environment, although temperature had some influence. The balance among these three processes results in an optimized supersaturation ratio for a controlled growth of iron-germanium nanowires.

At regular growth pressure (atmospheric pressure), the iron chloride could be decomposed at 550 °C, evidenced by the large amounts of iron-germanium compounds condensed on the substrate as micro-structures. To achieve the low supersaturation ratio needed to fulfill nanowire growth, high Ar pressure was applied to suppress the reaction speed and keep those reactions going moderately. When the pressure reached 1000 Torr, straight and long nanowires (200 nm in diameter and 10 µm in length) started to aggregate around the corner area of the germanium substrate, which may be the result of Ar pressure fluctuations near edges of the substrate.

**Figure 1:**
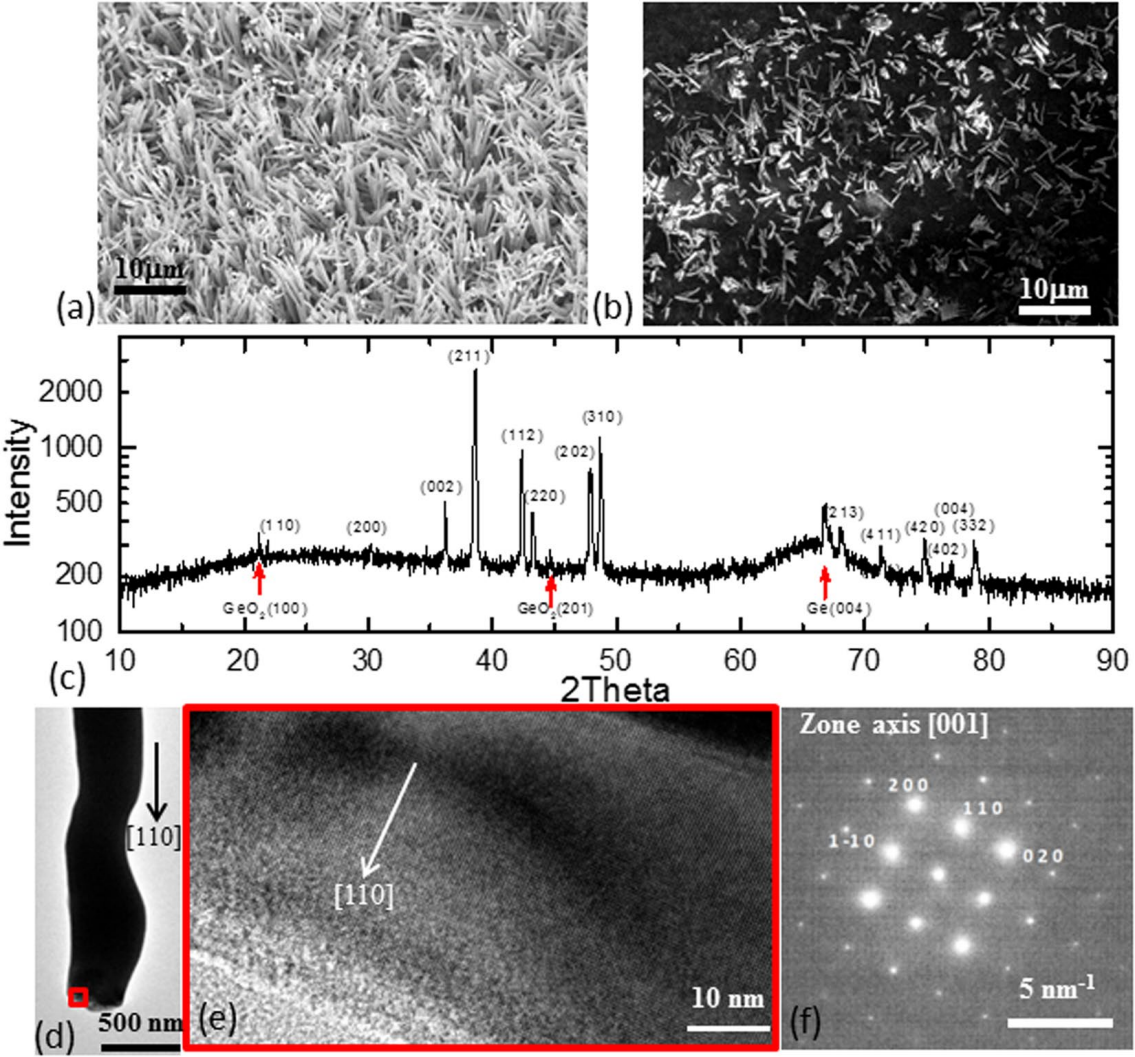
Structure of the nanowires. (**a**) SEM image of nanowires on Ge substrate, shows majority of the wires were vertically grown on the substrate. (**b**) SEM image of nanowires after transferred to carbon tape, shows wire were aligned on the tape. (**c**) X-ray diffraction pattern measured from the nanowires on Ge wafer, matches FeGe_2_ as indexed in the figure. Red arrows mark the Ge (004) peak of the substrate and impurity peaks identified as GeO_2_ (100) and (201). (**d**) and (**e**) TEM images from single nanowire. (**e**) is the zoom in of the red rectangle area in (**d**). (**f**) The electron diffraction pattern of (**e**), it identified that the structure matches space group I4/mcm and long axis direction of nanowires is along [110].

By increasing the pressure to 2400 Torr, grass-like vertical nanowires of approximately 100–300 nm diameter and 5–10 μm length (Fig. 1(a)) are spread across the substrate with a buffer layer about 3–10 μm in between. The crystal structure of nanowires was decided and verified by transmission electron microscopy (TEM), X-ray diffraction (XRD) and energy-dispersive X-ray spectroscopy (EDX) as shown in Fig. 1. Scanning the whole sample (nanowires, buffer layer and substrate) with XRD, all peaks except the three red-arrow marked peaks match FeGe_2_ (Fig. 1(c)) (space group I4/mcm) with lattice constant a = b = 5.899 Å, c = 4.941 Å. The three extra peaks are identified as Ge (004) at 66°, GeO_2_ (100) at 21° and (201) at 45°. So it is confirmed that nanowires and the buffer layer film are both FeGe_2_. Several nanowires were chosen from random locations of the substrate for structural verification using TEM (Fig. 1(d–f)). The nanowires were all found to be single crystals of tetragonal C16 FeGe_2_ structure. The long axis of nanowire is indicated as [110]. EDX measurements were done on tape tap-transferred individual nanowires. The EDX measurement shows uniform compositions throughout the wires, the average of the Fe/Ge composition ratio of multiple measurements obtained from the spectrum was 5.35 ± 1.18 and 7.37 ± 2.13. We believe the growing process is vapor-solid growth rather than vapor-liquid-solid growth, since our preparation process did not involve any catalyst as evidenced by TEM images of pure FeGe_2_ crystalline structure throughout the whole wire. In vapor-solid growth, the growth temperature and the supersaturation ratio (depends on the vapor pressure of precursors) dominate the morphology of the products^32–35^.

**Figure 2:**
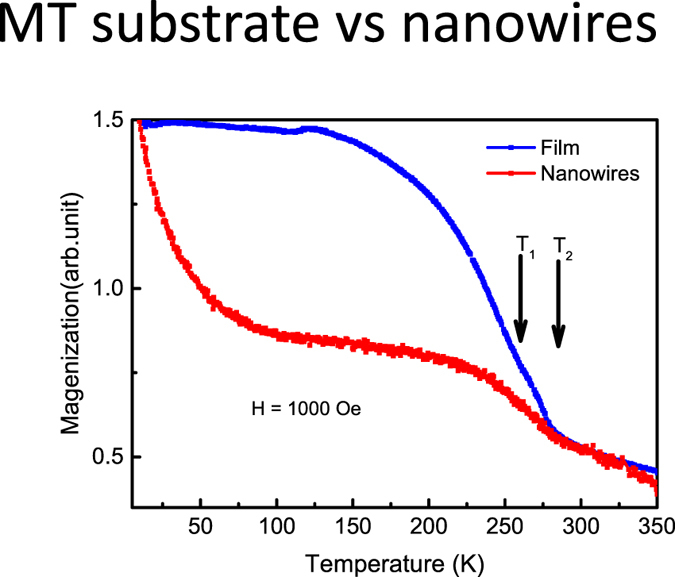
Comparison of the magnetization of film and nanowires. Arrows marked the two transitions near 260 K and 290 K. Both curves were measured at 1000 Oe.

It is interesting to compare the magnetic behavior of both nanowires and films, especially when the dimensionality effect starts to play a role^36^. Figure 2 shows normalized magnetizations vs. temperature for both samples. Note that the film sample is the buffer layer FeGe_2_ on Ge wafer after peeling off most of the nanowires, while the nanowires sample is a wire-only sample tape-transferred to a clean silicon substrate. The film sample shows subtle but visible (marked with arrows) magnetic transitions at around 290 K and 260 K, which are similar to bulk FeGe_2_. However, the magnetization data from the nanowires show vast differences. Under 1000 Oe external field, the magnetizations continue to increase all the way to 5 K.

**Figure 3:**
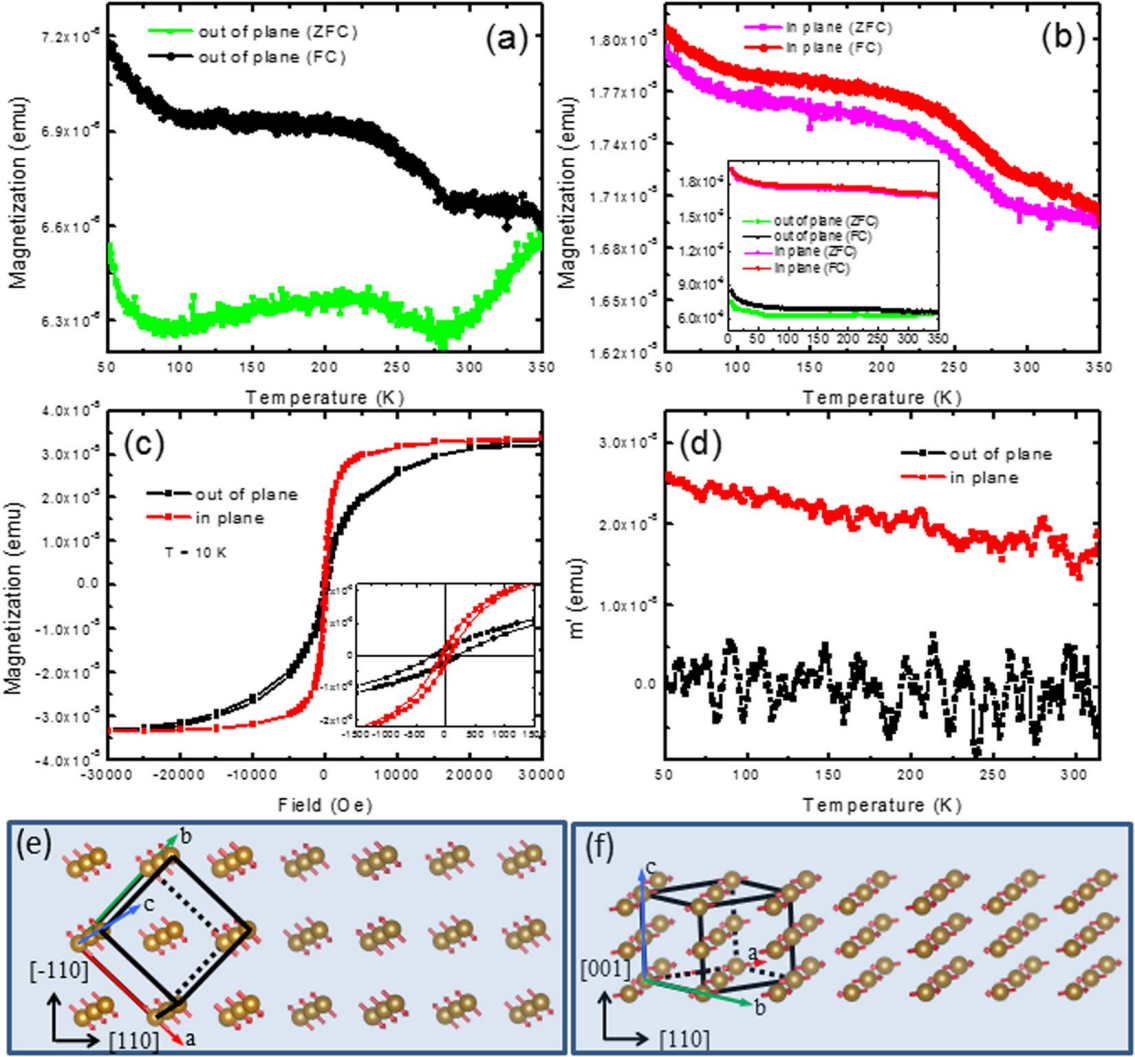
Anisotropic magnetic properties of FeGe_2_ nanowires. Temperature-dependent ZFC and FC magnetic moments when magnetic field was applied at out-of-plane direction (**a**) and in-plane direction (**b**), H = 1000 Oe. The inset of (**b**) shows the direct comparison of the out-of-plane and in-plane ZFC and FC data. (**c**) Field-dependent magnetization at 10 K for out-of-plane and in-plane, the inset is the zoom in the magnetization. (**d**) The initial ac susceptibilities under 0 field for out-of-plane and in-plane. (**e**) and (**f**) The schematic drawing of the relation among the two structure models of FeGe_2_, growth direction of the nanowires and the spin alignment of Fe atoms, with short axis in [−110] and [001] directions.

The detailed magnetic properties measured from the nanowires sample (described in experimental session) are shown in Fig. 3. The direct comparison of the out-of-plane (the magnetic field was applied perpendicular to the Si wafer) and in-plane (field parallel to the wafer) magnetizations shows strong anisotropic behavior. In zero field cool measurement (ZFC), the nanowires were cooled first without a field, then warmed and measured with applied field, however in field cool (FC), the nanowires were cooled and measured at the same time with applied external field. The FC magnetizations were measured with 1000 Oe field applied out-of-plane (Fig. 3(a)) and in-plane (Fig. 3(b)), they share the similar trend as described in Fig. 2. However, the ZFC curves are very different. There are several slope changes on the out-of-plane ZFC curve in Fig. 3(a), the details will be discussed later. For quantitative comparison, ZFC and FC curves in the two directions are plotted together as an inset of Fig. 3(b). The out-of-plane magnetization is much harder than in-plane magnetization. This is consistent with the field dependent magnetizations for both directions at 10 K shown in Fig. 3(c), which show a square-like magnetization for the in-plane magnetization, while the out-of-plane magnetization increases gradually all the way up to 30000 Oe. From the inset in Fig. 3(c), the remanence magnetization along the two directions are almost the same (Mr/Ms = 0.07), but the coercivities are slightly different, 70 Oe vs 205 Oe. The initial ac susceptibilities (under zero field) in both directions are shown in Fig. 3(d). While the in-plane ac susceptibility shows a sizable value and a reasonable rate of increase when the temperature is decreased, the out-of-plane data is very small, within the noise level of the equipment. This “easier” magnetization in-plane cannot be understood by the simple shape anisotropy effect of the wires.

The above anisotropic results indicate that the spin coupling in the nanowires is intrinsically different along the long axis (axial direction along the nanowire long axis [110]) and short axis (radial direction of the nanowire). As shown in Fig. 1(b), majority of the wires are aligned on the tape plane, the in-plane magnetization (magnetic field applied parallel to the tape) reflects the azimuthal average contribution of the long axis of all the wires on the tape plane, while the out-of-plane magnetization (field perpendicular to the tape) is the sum of the magnetization of the short axis of all wires. For bulk FeGe_2_, at zero or low field, the propagation vector of the SDW is parallel to [100], the spins lie in the basal plane and are ferromagnetically aligned along the [001] direction (*c* axis)^18, 23, 24^. Assuming the same magnetic structure persist in the nanowires form, along the wire long axis ([110] direction), antiferromagnetic incommensurate or commensurate SDW states form. But along the wire short axis, there are two inequivalent short axis directions, [−110] and [001]. Shown in Fig. 3(e) and (f) are the relative relation between the two magnetic structures model of FeGe_2_ and the geometry of the nanowires. Along [001] direction, Fe spins order ferromagnetically while along [−110] direction antiferromagnetically. Although it is difficult to make statements about [001] ordering versus [1–10] ordering, the anisotropic magnetization indicates the existence of some preference. It is interesting to note that the strong anisotropic behavior was also observed using inelastic neutron scattering to measure the spin dynamics of bulk FeGe_2_^22, 37^, as a result of the quasi-one-dimensional nature. The formation of nanowire arrays and the size limitation of the wires in the short axis direction may largely enhance the anisotropic magnetization in this system, or even possibly stabilize ferromagnetic order as predicted by ab initio density functional theory in FeGe_2_ thin film system^38, 39^. To further clarify the orientation preference, it is necessary to align an ensemble of wires along the long axis, either grow the materials under external magnetic field or using dielectrophoresis or solution based methods such as floating evaporative self-assembly^40^.

**Figure 4:**
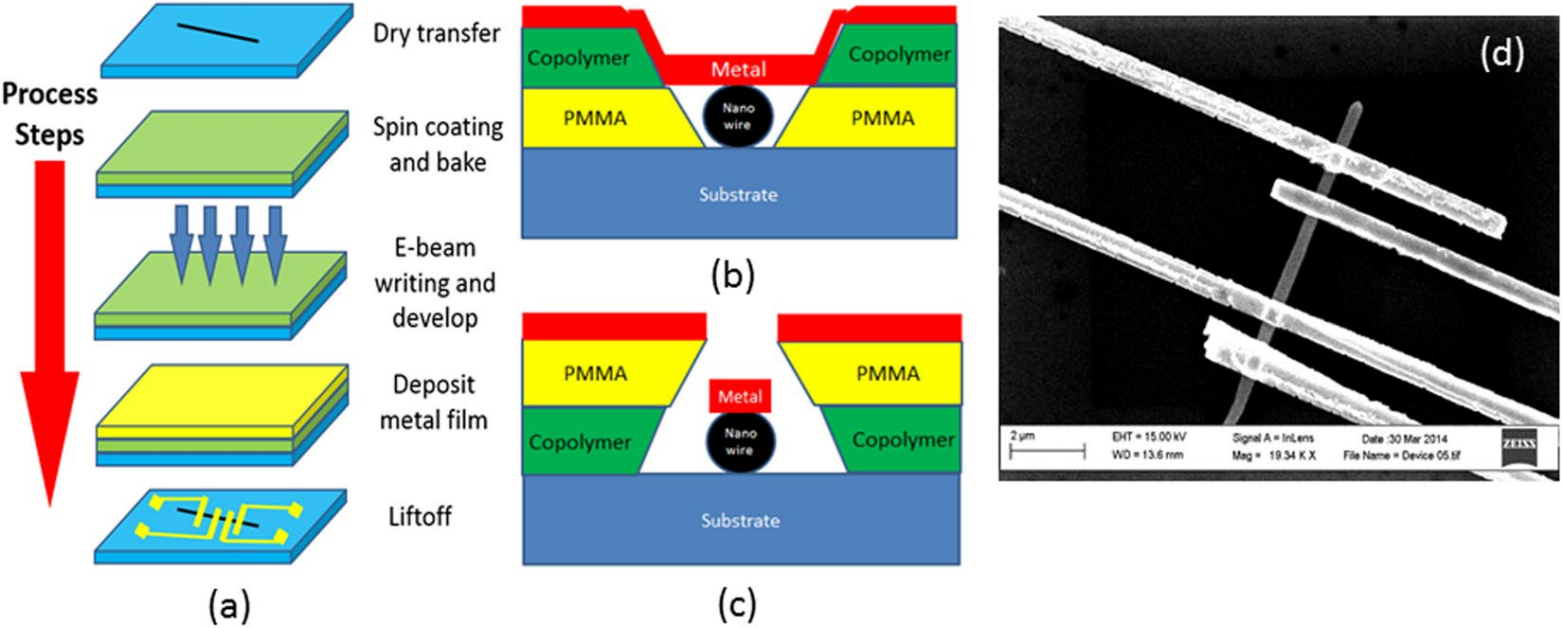
Fabricating process of the single wire devices. (**a**) Process steps for fabricating a single nanowire into four electrodes devices via e-beam lithography. Double layer configuration (**b**) with copolymer layer (slower develop speed) on the top formed an inverted trapezoid structure and (**c**) with PMMA layer (faster develop speed) on the top formed a trapezoid structure. (**d**) SEM image of a successful prepared four probe device.

To further explore the dimensionality effect, electronic transport was measured on a single FeGe_2_ nanowire with width and length approximately 600 nm and 10 µm, respectively. Figure 4 shows the processes for making those 4-probe single wire devices. As shown in Fig. 4(a), firstly, nanowires were dry transferred into acetone, then a drop of the solution with dispersed nanowires was transferred to a piece of silicon wafer, which was pre-coated with 300 nm of silicon oxide and pre-patterned with gold markers to facilitate locating of the nanowire during the lithography process. A spin coater was used to cover the wafer with PMMA and copolymer (e-beam resist). After coating, the wafer was baked at 100–200 °C for 20 seconds. The electrode contacts were predesigned case-by-case using Layout software and applied using a JEOL 9300FS 100 kV Electron Beam Lithography System to read and expose the chosen areas. After exposing, MIBK + IPA and isopropanol were used to develop the pattern. After that, 50 nm Cr and then 180 nm Au were deposited using e-beam evaporation onto the substrate. Finally, metal films in the unexposed areas were removed by acetone, and a liftoff strip.

We found that it is difficult to fabricate the devices via a single layer of PMMA. The problem came from the large width (height) of the nanowires compared to the thickness of the PMMA layer. As a result, the metallic layers on the unexposed PMMA area were easily connected to the electrodes making removal of selected regions difficult. To solve this problem, two improvements were employed: optimizing the dose of the electron beam; and utilizing a double layer geometry. Dose is defined as the electric charge (D = I_beam_*T_dwell_) exposed to the surface per unit area. I_beam_ is the beam current and T_dwell_ is the time for exposure. The dose of 1300 μC/cm^2^ was used for the best lithography result. Double layers (copolymer and PMMA) were used to increase the resist’s total thickness (lithography depth). Two geometries of these two layers were compared. The stacking sequence of the layers is important as the copolymer was much more sensitive to the e-beam and developed faster than the PMMA (Fig. 4(b) and (c)). In the design in Fig. 4(b), the copolymer will form a wider feature than PMMA, leading to the attachment of the electrodes to the metal film on the surface. In the design in Fig. 4(c), the slowly developed PMMA layer prevents the gold layer from attaching. The application of saw-tooth shapes totally alleviated all attachment issues. A SEM image of one of the devices is shown in Fig. 4(d).

**Figure 5:**
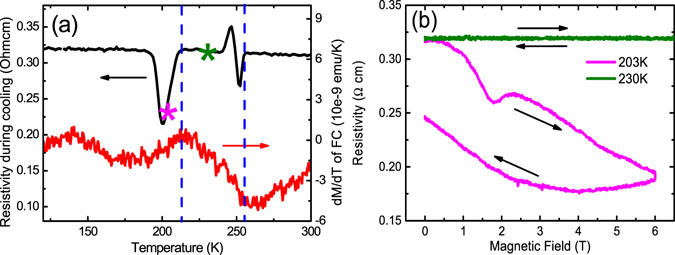
Electric transport of single wire. (**a**) Resistivity result measured during cooling with magnetic field of 60000 Oe applied perpendicular to the device plane, showing two anomalies at approximately 250 K and 200 K (left y axis), in comparison with dM/dT of FC magnetization (right y axis). Blue lines are for eye-guide. (**b**) Field dependent resistivity measured at 203 K and 230 K (temperatures marked with stars in (**a**)). The arrows show the field hysteresis.

Once isolated for 4-probe transport measurements, individual nanowires were found to present interesting anomalies in resistive behavior. Figure 5(a) shows the temperature dependent resistivity of one such device measured during cooling under a 6 T magnetic field applied perpendicular to the device plane. The resistivity data shows two large features at approximately 250 K and 200 K. The shape and height of the resistive peaks and valleys and their onset temperatures show slight deviations from device to device, but the two-step transition feature is quite similar. Detailed resistivity vs magnetic field scans taken within a valley-site window at a temperature of 203 K and at an arbitrarily selected off-feature temperature of 230 K between the two valley-site temperature windows are shown in Fig. 5(b). We observe that there is no observable field dependent resistivity change when the temperature is outside of the valley-site windows. However, a strong magnetic field sensitivity evolves within the valley-site window at 203 K and is expressed in the form of a large change to resistivity and pronounced hysteresis. Similar but weaker field-dependent resistivity and hysteresis was observed at the 250 K valley-site feature while all measured temperatures outside of these windows showed no significant response to applied magnetic field. Interestingly, the onsets of the valley-site temperature windows closely align to the observed magnetic transitions of the FeGe_2_. Specifically, we see in Fig. 5(a) that the first derivative of the out-of-plane FC magnetization from Fig. 3(a) coincidently correlates to the onset of the observed resistivity features. Surface oxidation of the nanowires might be a concern for the transport measurement, but this abnormal behavior was not observed in any other systems, including other material systems with Fe or oxides of Fe. Furthermore, the observable oxidation layer is GeO_**2**_ as evidenced by XRD, but GeO_**2**_ is diamagnetism, so we attribute the magnetic related anomalous resistance behavior to the dimensionality effect of FeGe2 wires.

For bulk FeGe_2_, there are two magnetic phase transitions at 289 K and at 263 K^18, 23, 24^. The magnetic phase transitions of FeGe_2_ under external field are quite complicated based on the experimental result and theoretical calculations because of the strong coupling between magnetic structure and crystal structure. Under external magnetic field, there exist several states and phase boundaries^23^. The observed resistivity features in our current data are not sufficient to extract the anomalies’ exact mechanisms; however, they do allow us to offer important speculation to guide future studies. We anticipate that for nanowires, the slope changes in the first derivative of magnetization correspond to the two magnetic phase transitions, which unsurprisingly occur at lower temperatures in the confined systems due to dimensionality effects. Thus, we assume that the higher temperature resistivity feature relates to the boundary of the paramagnetic to incommensurate SDW transition, while the lower temperature resistivity feature relates to the boundary between incommensurate SDW and commensurate SDW transition. This is consistent with claims that a magnetic super-zone state forms at the transition boundaries in bulk FeGe_2_ which is known to drive resistive changes arising from increased scattering of the charge carriers^24^. In the nanowire form, with the diameter of the nanowires being comparable to the size of magnetic domains, it is thus reasonable that any changes associated with the spin flips and domain walls give rise to much more pronounced features in the resistivity around the transition temperatures^41^.

## Conclusion

Single-crystal FeGe_2_ nanowires were synthesized on germanium wafer by chemical vapor deposition method. Global magnetic measurements of FeGe_2_ nanowire arrays show strong anisotropic behavior: temperature dependent magnetization and field dependent magnetization and ac susceptibility show the mixture of ferromagnetism and antiferromagnetism. Single wire devices with four electrodes were successfully fabricated using e-beam lithography with dose correction and double layer geometry. The resistivity of isolated nanowires shows two resistive anomalies around 250 K and 200 K which correspond to abnormal resistivity changes at the magnetic transitions of the SDW state in bulk FeGe_2_, but are greatly enhanced by dimensionality effects under confinement. The ability to control and isolate these dimensionality effects in an antiferromagnetic system thus offers a tantalizing path forward in understanding and creating novel spin-dependent applications in the emerging fields of antiferromagnetic storage, resistive switching, and spintronics.

## Methods

### Chemical vapor deposition

We used chemical vapor deposition to grow the FeGe_2_ nanowires. The system was set up based on a tube furnace with a mechanical pump and gas valve together to control temperature and pressure^11^. Ar was used as the carrier gas, the flow rate was software controlled. The system allows precise control of growth temperature, gas flow rate, gas pressure, and distance from substrate to precursor. Iron (III) chloride FeCl_3_ was used as the precursor, and a germanium (001) wafer was used as the growth substrate. Precursors and substrate were put in the silica tube locating 1.5 cm from the opening and 6 cm away from the tube center, respectively. Iron chloride was selected as the iron source due to its relatively low evaporation, decomposition, and reaction temperatures.

### Dry Transferred

The nanowires were transferred to silicon substrates for magnetic measurements by gently applying Kapton tape to the Ge wafer to pick up many nanowires; the tape was then affixed to a clean silicon substrate. The tape and clean silicon substrate were checked and confirmed in advance to be diamagnetic.

### Phase Identification

The presence of the nanowires on the tape was verified using Zeiss Merlin VP scanning electron microscopy (SEM) and energy dispersed x-ray spectroscopy mapping (EDX). Nanowires were also examined using FEI-Titan transmission electron microscopy (TEM) to verify the structure from the diffraction pattern. X-ray diffraction was used to scan the whole sample to confirm the primary phase of the samples.

### Magnetic Measurements

Magnetic properties of the synthesized material were characterized with a Quantum Design Magnetic Property Measurement System (MPMS)^31, 35^. Measurements were made both with and without Ge wafers. The wafers and substrates were mounted either perpendicular to the magnetic field (out-of-plane magnetization) or parallel to the field (in-plane magnetization). The magnetic data represent the global properties of samples.

### Device Fabrication and resistance measured

In order to investigate the electric transport behavior of individual FeGe_2_ nanowires, single-wire devices were fabricated using e-beam lithography. Electrical transport measurements were carried out using a Quantum Design Physical Property Measurement System (PPMS-9T). Device substrates were mounted to a chip carrier surface with silver colloidal paint, and 4 contact electrodes were wire bonded to the chip carrier. Resistivity measurements with and without magnetic field were accomplished using standard DC techniques with a Keithley 2400 Source Meter. Special care was taken during the handling of the nanowire devices for the electrical transport measurements, as the devices were easily destroyed by static electric shock or even during the vacuum pump down. During the measurements, the applied current was kept below 1 µA to ensure device integrity during the measurement.
